# 

**Published:** 1884-02

**Authors:** 


ORIGINAL COMMUNICATIONS:
Fermentation in the Human Mouth; Its relation to Caries of the Teeth. W. D. Miller .......... 57
System as Applied to Instruments and Books. W. St. George Elliott .......... 65
Dental Notes in Different Parts of the World .......... 71
A Case of Fracture of the Inferior Jaw. T. B. Gunning .......... 77
Remarks upon Diseases of the Antrum. Frank Abbott .......... 79
REPORTS OF SOCIETY MEETINGS:
Central Association of Northern New Jersey .......... 85
CORRESPONDENCE:
From Rome .......... 93
The "M. D. S." .......... 94
EDITORIAL:
Chloride of Zinc .......... 95
A National Dental Society .......... 97
Fermentation in the Human Mouth .......... 100
Gum Guillotine Forceps *vs.* Gum Scissors .......... 101
Woman .......... 101
The Pot *vs.* The Kettle .......... 102
Bibliographical .......... 102
Celluloid .......... 103
Celluloid Handles for Instruments .......... 103
A Change .......... 103
OBITUARY:
Dr. W. H. Long .......... 104
CURRENT NEWS AND OPINION:
The Teeth of the Future .......... 105
Therapeutic Agents for the Promotion of Osseous Development .......... 106
Zonular Cataract with Characteristic Teeth .......... 106
Units for Measurements .......... 107
University of Buffalo .......... 108
Naphthaline as a Disinfectant .......... 108
Transactions (N. Y.) for 1882–83 .......... 109
Russian Teeth .......... 109
Dissolution .......... 110
The American Monthly Microscopical Journal .......... 110
Celluloid Disks .......... 110
Removed .......... 110
For 1884 .......... 111
Value of Artificial Respiration .......... 111
Removal .......... 111

